# High-resolution Microbiome Analyses of Nine Psyllid Species of the Family Triozidae Identified Previously Unrecognized but Major Bacterial Populations, including *Liberibacter* and *Wolbachia* of Supergroup O

**DOI:** 10.1264/jsme2.ME22078

**Published:** 2022-12-06

**Authors:** Atsushi Nakabachi, Hiromitsu Inoue, Yuu Hirose

**Affiliations:** 1 Electronics-Inspired Interdisciplinary Research Institute (EIIRIS), Toyohashi University of Technology, 1–1 Hibarigaoka, Tempaku, Toyohashi, Aichi 441–8580, Japan; 2 Department of Applied Chemistry and Life Sciences, Toyohashi University of Technology, 1–1 Hibarigaoka, Tempaku, Toyohashi, Aichi 441–8580, Japan; 3 Institute for Plant Protection, National Agriculture and Food Research Organization, Higashihiroshima, Hiroshima 739–2494, Japan

**Keywords:** *Liberibacter*, *Wolbachia* supergroup O, *Diplorickettsia*, *Serratia symbiotica*, *Carnimonas*

## Abstract

Psyllids (Hemiptera: Sternorrhyncha: Psylloidea) are plant sap-sucking insects that include important agricultural pests. To obtain insights into the ecological and evolutionary behaviors of microbes, including plant pathogens, in Psylloidea, high-resolution ana­lyses of the microbiomes of nine psyllid species belonging to the family Triozidae were performed using high-throughput amplicon sequencing of the 16S rRNA gene. Analyses identified various bacterial populations, showing that all nine psyllids have at least one secondary symbiont, along with the primary symbiont “*Candidatus* Carsonella ruddii” (*Gammaproteobacteria*: *Oceanospirillales*: *Halomonadaceae*). The majority of the secondary symbionts were gammaproteobacteria, particularly those of the order *Enterobacterales*, which included *Arsenophonus* and *Serratia symbiotica*, a bacterium formerly recognized only as a secondary symbiont of aphids (Hemiptera: Sternorrhyncha: Aphidoidea). The non-*Enterobacterales* gammaproteobacteria identified in the present study were *Diplorickettsia* (*Diplorickettsiales*: *Diplorickettsiaceae*), a potential human pathogen, and *Carnimonas* (*Oceanospirillales*: *Halomonadaceae*), a lineage detected for the first time in Psylloidea. Regarding alphaproteobacteria, the potential plant pathogen “*Ca.* Liberibacter europaeus” (*Rhizobiales*: *Rhizobiaceae*) was detected for the first time in *Epitrioza yasumatsui*, which feeds on the Japanese silverberry *Elaeagnus umbellata* (Elaeagnaceae), an aggressive invasive plant in the United States and Europe. Besides the detection of *Wolbachia* (*Rickettsiales*: *Anaplasmataceae*) of supergroup B in three psyllid species, a lineage belonging to supergroup O was identified for the first time in Psylloidea. These results suggest the rampant transfer of bacterial symbionts among animals and plants, thereby providing deeper insights into the evolution of interkingdom interactions among multicellular organisms and bacteria, which will facilitate the control of pest psyllids.

Psyllids (Hemiptera: Sternorrhyncha: Psylloidea) are plant sap-sucking insects comprising approximately 4,000 described species worldwide (Burckhardt *et al.*, 2021). Since they feed exclusively on phloem sap, some species transmit plant pathogens, including “*Candidatus* Liberibacter spp.” (*Alphaproteobacteria*: *Rhizobiales*), making them notorious agricultural pests (Grafton-Cardwell *et al.*, 2013; Mora *et al.*, 2021). Furthermore, phloem sap is deficient in some essential nutrients (Ziegler *et al.*, 1975); therefore, psyllids depend on vertically transmitted bacterial mutualists to compensate for these deficiencies. They typically harbor two distinct symbionts (Thao *et al.*, 2000a; Spaulding and von Dohlen, 2001; Sloan and Moran, 2012; Nakabachi *et al.*, 2013b, 2020a, 2020b, 2022; Arp *et al.*, 2014; Hall *et al.*, 2016; Morrow *et al.*, 2017) within an organ called the bacteriome (Nakabachi *et al.*, 2010). “*Ca.* Carsonella ruddii” (*Gammaproteobacteria*: *Oceanospirillales*) (Thao *et al.*, 2000b; Spaulding and von Dohlen, 2001; Sloan and Moran, 2012; Nakabachi *et al.*, 2013b, 2020a, 2020b, 2022; Arp *et al.*, 2014; Hall *et al.*, 2016; Morrow *et al.*, 2017; Nakabachi and Moran, 2022) is the ‘primary symbiont’ that provides the host with essential amino acids (Nakabachi *et al.*, 2006, 2013b, 2020b; Sloan and Moran, 2012). Molecular phylogenetic ana­lyses demonstrated cospeciation between psyllids and *Carsonella*, resulting from the single acquisition of a *Carsonella* ancestor by a psyllid common ancestor and its subsequent stable vertical transmission (Thao *et al.*, 2000b; Spaulding and von Dohlen, 2001; Hall *et al.*, 2016; Nakabachi *et al.*, 2020a, 2022). Another bacterial lineage housed in the bacteriome is categorized as a ‘secondary symbiont’, which is phylogenetically diverse among psyllid species and genera, suggesting multiple infections and replacements during the evolution of Psylloidea (Thao *et al.*, 2000a; Spaulding and von Dohlen, 2001; Sloan and Moran, 2012; Hall *et al.*, 2016; Morrow *et al.*, 2017; Nakabachi *et al.*, 2020a, 2022). Secondary symbionts in various insects range from parasites to facultative mutualists (Moran *et al.*, 2008), whereas those in the psyllid bacteriome analyzed to date invariably exhibit the features of obligate mutualists (Sloan and Moran, 2012; Nakabachi *et al.*, 2013b, 2020b; Hall *et al.*, 2016; Morrow *et al.*, 2017). Since these features are characteristic of nutritional symbionts (Moran *et al.*, 2008), secondary symbionts in the psyllid bacteriome are generally considered to have a nutritional basis (Spaulding and von Dohlen, 2001; Sloan and Moran, 2012; Morrow *et al.*, 2017). A unique exception is “*Ca.* Profftella armatura” (*Gammaproteobacteria*: *Burkholderiales*) found in psyllids of the genus *Diaphorina* (Psyllidae: Diaphorininae) (Nakabachi *et al.*, 2013b, 2020a, 2020b; Dan *et al.*, 2017), the main role of which appears to be protecting the holobiont (the host plus symbionts) from natural enemies‍ ‍(Nakabachi *et al.*, 2013b, 2020b; Nakabachi and Fujikami, 2019; Nakabachi and Okamura, 2019; Yamada *et‍ ‍al.*, 2019; Tanabe *et al.*, 2022). In addition to these bacteriome-associated obligate mutualists, psyllids may harbor various secondary symbionts of a facultative nature, including *Wolbachia* (*Alphaproteobacteria*: *Rickettsiales*), *Rickettsia* (*Alphaproteobacteria*: *Rickettsiales*), *Rickettsiella* (*Gammaproteobacteria*: *Diplorickettsiales*), and *Diplorickettsia* (*Gammaproteobacteria*: *Diplorickettsiales*), which cause systemic infections in host insects (Spaulding and von Dohlen, 2001; Sloan and Moran, 2012; Arp *et al.*, 2014; Chu *et al.*, 2016; Jain *et al.*, 2017; Kruse *et al.*, 2017; Morrow *et al.*, 2017; Chu *et al.*, 2019; Nakabachi *et al.*, 2020a, 2022). Moreover, similar to other hemipteran insects (Nakabachi and Ishikawa, 1997, 1999; Nakabachi *et al.*, 2003, 2005, 2014; Moran *et al.*, 2008; Kikuchi, 2009; Nikoh and Nakabachi, 2009; Tamborindeguy *et al.*, 2010; Shigenobu *et al.*, 2010; Nakabachi, 2015; Uchi *et al.*, 2019), recent studies revealed that not only interactions between host psyllids and symbiotic microbes, including those associated with the bacteriome, facultative symbionts, and plant pathogens (Nakabachi *et al.*, 2006, 2010, 2013a; Sloan *et al.*, 2014; Dan *et al.*, 2017; Nakabachi and Fujikami, 2019), but also interactions among these bacterial populations are important for psyllid biology and host plant pathology (Nakabachi *et al.*, 2013a, 2020a, 2022; Chu *et al.*, 2016; Dan *et al.*, 2017; Jain *et al.*, 2017; Kruse *et al.*, 2017; Chu *et al.*, 2019; Killiny, 2022; Tanabe *et al.*, 2022). Therefore, elucidating the microbiomes in various psyllid lineages, which reflect the ecological and evolutionary behaviors of bacterial populations in Psylloidea, will guide strategies to better control pest species.

Psyllids are currently classified into seven extant families: Aphalaridae, Calophyidae, Carsidaridae, Liviidae, Mastigimatidae, Psyllidae, and Triozidae (Burckhardt *et al.*, 2021). After Psyllidae, Triozidae is the second largest family consisting of 70 genera with more than 1,000 species (Burckhardt *et al.*, 2021). This family encompasses important agricultural pests, including the African citrus psyllid *Trioza erytreae*, the potato/tomato psyllid *Bactericera cockerelli*, and the carrot psyllids *Bactericera trigonica* and *Trioza apicalis* (Grafton-Cardwell *et al.*, 2013; Mora *et al.*, 2021). *T. erytreae* transmits “*Ca.* Liberibacter africanus” (*C*Laf, *Alphaproteobacteria*: *Rhizobiales*), which causes huanglongbing or citrus greening, the most destructive disease of citrus plants (Rutaceae) (Grafton-Cardwell *et al.*, 2013). *B. cockerelli*, *B. trigonica*, and *T. apicalis* transmit “*Ca.* Liberibacter solanacearum” (*C*Lso), the pathogen of severe diseases in important agricultural crops belonging to the families Solanaceae and Apiaceae, including potato, tomato, pepper, tobacco, carrot, and celery (Mora *et al.*, 2021). Although several high-throughput amplicon-sequencing ana­lyses have been performed on the microbiomes of Triozidae, the target psyllids were biased towards the most devastating pest *B. cockerelli* (Nachappa *et al.*, 2011; Arp *et al.*, 2014; Morrow *et al.*, 2017). A recent study analyzed 20 Triozidae species (Kwak *et al.*, 2021); however, the ana­lysis was performed using 1) limited numbers of specimens; 12 species were examined with only a single individual, making the ana­lysis less reliable, 2) primers unsuitable to detect symbionts with AT-rich genomes, and 3) an analytical method that clusters sequence reads with a similarity threshold of 97%, resulting in an ana­lysis with a lower resolution.

In the present study, we performed amplicon ana­lyses based on Illumina sequencing of 16S rRNA genes to assess the microbiomes of nine Triozidae species collected in Japan (Table 1) using 1) ten pooled individuals for each species, 2) primers to detect genomes with a wider variety of GC contents, and 3) a method to resolve sequence variants (SVs) down to the level of single-nucleotide differences.

## Materials and Methods

### Insects and DNA extraction

The adults of nine psyllid species belonging to the family Triozidae were collected from their host plants at various locations in Japan (Table 1). Insect samples were stored in acetone (*Epitrioza yasumatsui*, *Trioza cinnamomi*, and *Trioza machilicola*) or 99.5% ethanol (the other species) at –20°C until DNA extraction. After surface sterilization with ethanol, DNA was extracted from the whole bodies of pooled individuals of five adult males and five adult females of each psyllid species using the DNeasy Blood & Tissue Kit (Qiagen). The quality of the extracted DNA was assessed using a NanoDrop 2000c spectrophotometer (Thermo Fisher Scientific) and its quantity was evaluated using a Qubit 2.0 Fluorometer with the Qubit dsDNA HS Assay Kit (Thermo Fisher Scientific).

### Construction and sequencing of amplicon libraries

Bacterial populations in psyllids were analyzed using the MiSeq system (Illumina), as previously described (Nakabachi *et al.*, 2020a, 2022). In brief, amplicon PCR was performed using DNA extracted from psyllids, KAPA HiFi HotStart ReadyMix (KAPA Biosystems), and the primer set 16S_341Fmod (5′-TCGTCGGCAGCGTCAGATGTGTATAAGAGACAGYYTAMGGRNGGCWGCAG-3′) and 16S_805R (5′-GTCTCGTGGGCTCGGAGATGTGTATAAGAGACAGGACTACHVGGGTATCTAATCC-3′) targeting the V3 and V4 regions of the 16S rRNA gene. Both primers were based on the instructions by Illumina (Illumina, 2013), whereas 16S_341F was modified (underlined) with original CC, C, and G being replaced by the mixed bases YY (C or T), M (A or C), and R (A or G). This modification has been shown to increase the sensitivity of detecting symbionts with AT-rich genomes, including *Carsonella*, without reducing sensitivity for those with GC-rich genomes (Nakabachi *et al.*, 2022). Dual indices and Illumina sequencing adapters were attached to the amplicons with index PCR using Nextera XT Index Kit v2 (Illumina). The libraries were combined with PhiX Control v3 (Illumina), and 250 bp of both ends were sequenced on the MiSeq platform (Illumina) with MiSeq Reagent Kit v2 (500 cycles; Illumina).

### Computational ana­lysis of bacterial populations

Output sequences were imported into the QIIME2 platform (version 2022.2) (Bolyen *et al.*, 2019) and processed as previously described (Nakabachi *et al.*, 2020a, 2022). Briefly, after removing primer sequences, the denoising and joining of paired-end reads and the removal of low-quality or chimeric reads were performed using the dada2 plugin (Callahan *et al.*, 2016). The q2-feature-classifier plugin (Bokulich *et al.*, 2018) was trained using the V3 and V4 regions of the 16S rRNA gene sequences (Silva 138 SSURef NR99) (Glöckner *et al.*, 2017). Dereplicated amplicon reads were then classified, to which taxonomic information was assigned using the trained q2-feature-classifier. The sequence variants (SVs) obtained were manually checked by performing BLASTN searches against the National Center for Biotechnology Information non-redundant database (Camacho *et al.*, 2009).

### Phylogenetic ana­lysis of detected bacteria

Phylogenetic ana­lyses of SVs were performed as previously described (Nakabachi *et al.*, 2020a, 2022). In brief, after SVs were aligned to related sequences using SINA (v1.2.11) (Pruesse *et al.*, 2012), phylogenetic trees were inferred by the maximum likelihood (ML) method using RAxML (version 8.2.12) (Stamatakis, 2014). The GTR+Γ model was used without partitioning of the data matrix, with 1,000 bootstrap iterations (options -f a -m GTRGAMMA -# 1000).

### Data availability

Nucleotide sequence data are available in the DDBJ/EMBL/GenBank databases under the accession numbers DRR403084–DRR403092 (MiSeq output) and TAAC01000001–TAAC01000048 (dereplicated sequence variants).

## Results and Discussion

### All nine Triozidae species have *Carsonella* and at least one secondary symbiont

**Fig. 1.**
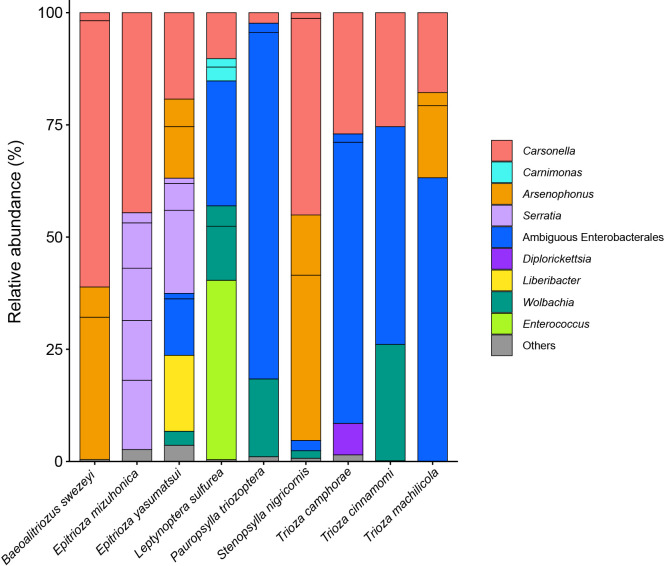
Composition of bacterial populations in psyllids of the family Triozidae. The relative abundance of Illumina reads belonging to the assigned bacterial taxa are shown.

**Fig. 2.**
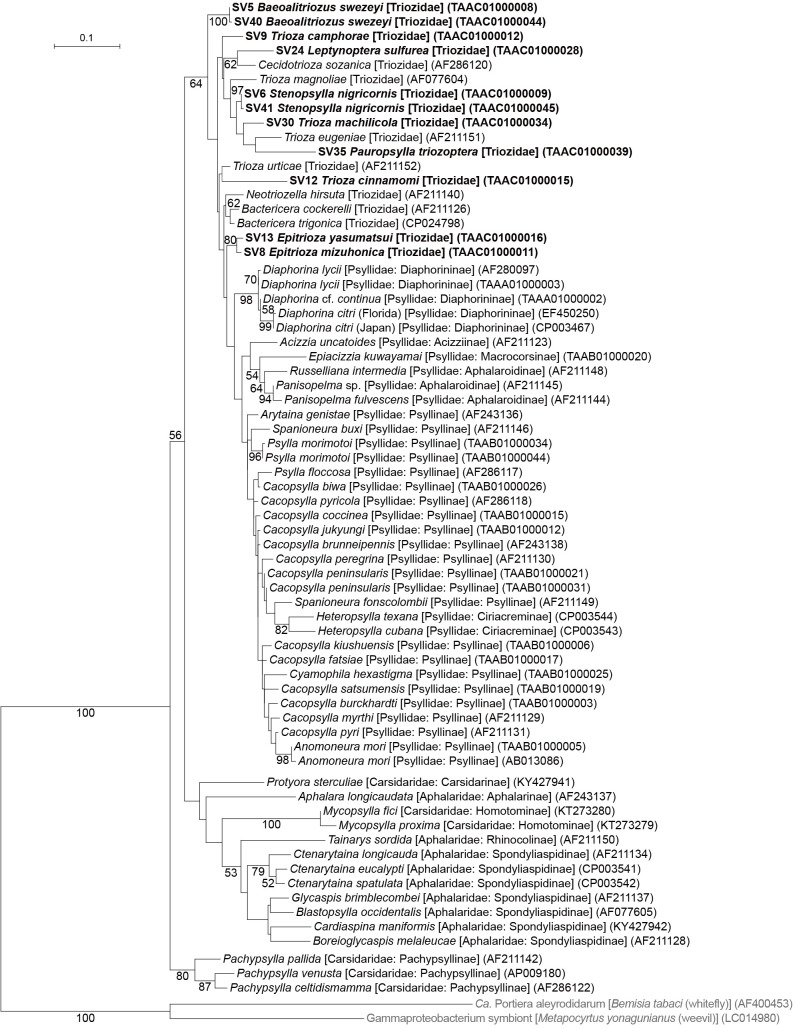
Maximum likelihood phylogram of *Carsonella*. A total of 427 unambiguously aligned nucleotide sites of 16S rRNA genes were subjected to the ana­lysis. On each branch, bootstrap support values of >50% are shown. Designations other than those for outgroups refer to psyllid hosts. Families and subfamilies (if applicable) of the host psyllids are shown in brackets. Sequences from this study are shown in bold. DDBJ/EMBL/GenBank accession numbers for sequences are provided in parentheses. The bar represents nucleotide substitutions per position. The outgroups were *Ca.* Portiera aleyrodidarum, the primary symbiont of the whitefly *Bemisia tabaci* (Hemiptera: Sternorrhyncha: Aleyrodoidea), and a gammaproteobacterium symbiont of the weevil *Metapocyrtus yonagunianus* (Coleoptera: Curculionidae).

MiSeq sequencing of amplicon libraries yielded 43,775–90,039 pairs of forward and reverse reads for the nine psyllid species. The denoising and joining of paired-end reads along with the removal of low-quality or chimeric reads resulted in 17,395–77,671 non-chimeric high-quality reads (Supplementary Table S1). The dereplication of these reads resulted in 206 independent sequence variants (SVs), among which 44 accounted for >1% of total reads (Supplementary Table S2). We focused on these 44 SVs because filtering with a threshold of 1% was shown to be among the most effective and accurate methods to remove potential contaminants (Karstens *et al.*, 2019). SVs with a relative abundance of less than 1% were collectively categorized as ‘others’ in Fig. 1, which accounted for 0% (*T. machilicola*)–3.6% (*E. yasumatsui*) of total reads in each psyllid species (Supplementary Table S2). Extremely simple bacterial communities of this type have been reported for sternorrhynchan insects with a bacteriome, including aphids, whiteflies, and other psyllid species (Nachappa *et al.*, 2011; Russell *et al.*, 2013; Jing *et al.*, 2014; Overholt *et al.*, 2015; Morrow *et al.*, 2017; Meng *et al.*, 2019; Nakabachi *et al.*, 2020a, 2022; Kwak *et al.*, 2021). Taxonomic classification by QIIME2 (Supplementary Table S2) followed by independent BLAST searches and phylogenetic ana­lyses showed that all nine psyllid species possess distinct lineages of *Carsonella* (Fig. 1). In the maximum likelihood (ML) tree, the *Carsonella* sequences identified in the present study formed a moderately supported clade (bootstrap: 64%) with those of psyllids belonging to the family Psyllidae (Fig. 2), which is consistent with the host psyllid phylogeny inferred by mitochondrial and nuclear data ana­lyses (Burckhardt *et al.*, 2021). Two types each of *Carsonella* sequences were observed in *Baeoalitriozus swezeyi* and *Stenopsylla nigricornis* (Fig. 1 and 2, Supplementary Table S2). In *B. swezeyi*, SV5 (59.4% of reads) and SV40 (1.8% of reads) were 99.8% identical (Supplementary Table S2). In *S. nigricornis*, SV6 (43.8% of reads) and SV41 (1.3% of reads) were also 99.8% identical (Supplementary Table S2). Although the dada2 plugin corrects sequencing errors during the denoising process (Callahan *et al.*, 2016), SV40 and SV41 may have been derived from PCR/sequencing errors because they accounted for only small percentages of the reads. Previous studies detected only a trace amount of *Carsonella* reads (Nachappa *et al.*, 2011; Morrow *et al.*, 2017; Kwak *et al.*, 2021), whereas the present study, which used primers with increased sensitivity to AT-rich symbiont genomes, detected a large percentage of *Carsonella* reads (Fig. 1 and Supplementary Table S2), more precisely reflecting actual populations (Nakabachi *et al.*, 2020a, 2022).

Besides *Carsonella*, all nine psyllids analyzed in the present study possessed at least one other symbiont (Fig. 1).

### *Leptynoptera sulfurea* has *Halomonadaceae* symbionts other than *Carsonella*

**Fig. 3.**
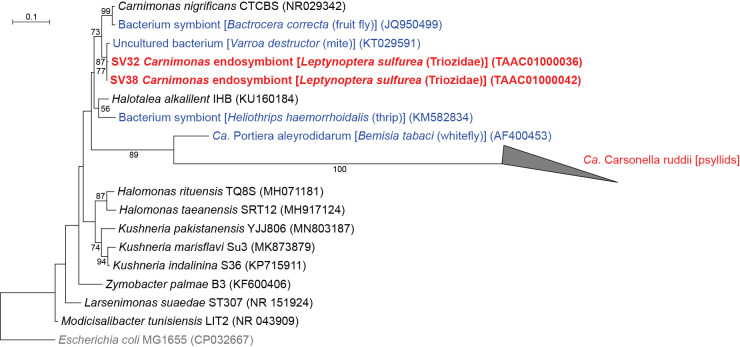
Maximum likelihood phylogram of bacteria belonging to *Halomonadaceae*. A total of 427 unambiguously aligned nucleotide sites of 16S rRNA genes were subjected to the ana­lysis. On each branch, bootstrap support values of >50% are shown. The scale bar indicates substitutions per site. The triangle represents the collapsed clade of *Carsonella* shown in Fig. 2. Regarding symbiotic bacteria, host organisms are shown in brackets. Symbionts of animals other than psyllids are shown in blue. Symbionts of psyllids are shown in red. Sequences from this study are shown in bold. DDBJ/EMBL/GenBank accession numbers are provided in parentheses. *Escherichia coli* was used as an outgroup.

QIIME2 assigned SV32 and SV38, which accounted for 3.1 and 1.9% of *L. sulfurea* reads, respectively, to *Carnimonas* (*Gammaproteobacteria*: *Oceanospirillales*: *Halomonadaceae*) (Fig. 1 and 3, Supplementary Table S2). These SVs were 97.9–99.5% identical to the sequences of *Carnimonas nigrificans* (NR029342), the type species of the genus, and its relatives isolated from arthropod hosts (KT029591 and JQ950499). In the ML tree, these sequences formed a moderately supported clade (bootstrap: 73%) (Fig. 3). Although *Carsonella* also belongs to this family (*Oceanospirillales*: *Halomonadaceae*) (Spaulding and von Dohlen, 2001; Sloan *et al.*, 2014), sequence identity between these SVs and *Carsonella* was low (<80%). Moreover, the clade of *Carnimonas* symbionts excluded a well-supported clade (bootstrap: 89%) of *Carsonella* and “*Ca.* Portiera aleyrodidarum”, the primary symbiont of whiteflies (Hemiptera: Sternorrhyncha: Aleyrodoidea) (Fig. 3), indicating that the lineages newly identified in the present study are distantly related to *Carsonella*.

### Various bacteria of *Enterobacterales* reside in Triozidae

**Fig. 4.**
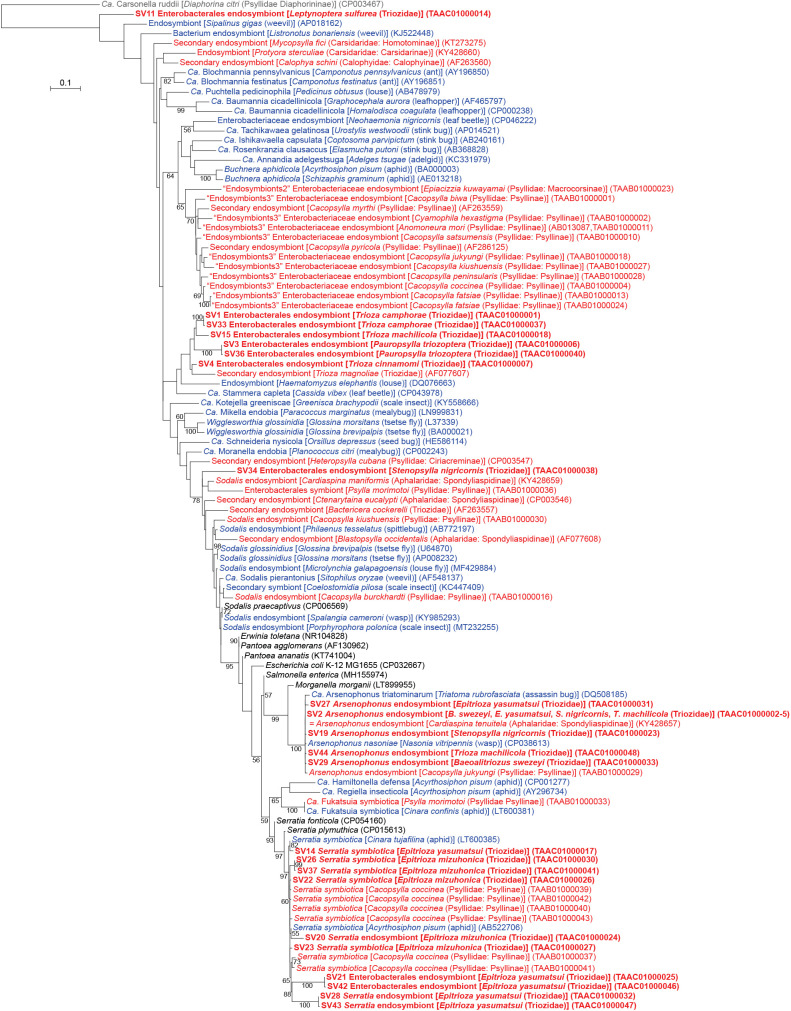
Maximum likelihood phylogram of *Enterobacterales*. A total of 427 unambiguously aligned nucleotide sites of 16S rRNA genes were subjected to the ana­lysis. On each branch, bootstrap support values of >50% are shown. The scale bar indicates substitutions per site. Regarding symbiotic bacteria, host organisms are shown in brackets. Symbionts of animals other than psyllids are shown in blue. Symbionts of psyllids are shown in red. Sequences from this study are shown in bold. DDBJ/EMBL/GenBank accession numbers are provided in parentheses. *Carsonella* was used as an outgroup.

Among the 44 main SVs obtained in the present study, 37 corresponded to gammaproteobacteria, among which 23 belonged to the order *Enterobacterales* (Supplementary Table S2). *Enterobacterales* is a group of bacteria that encompasses a large fraction of intimate insect symbionts, including those associated with the bacteriome (Moran *et al.*, 2008). *Enterobacterales* bacteria identified in the present study included *Arsenophonus*, *Serratia symbiotica*, and several lineages with ambiguous phylogenetic placements (Fig. 1 and 4, Supplementary Table S2).

### *Arsenophonus* symbionts

Five SVs corresponding to distinct *Arsenophonus* lineages were detected in four of the nine Triozidae species: *B. swezeyi*, *E. yasumatsui*, *S. nigricornis*, and *T. machilicola* (Fig. 1 and 4, Supplementary Table S2). SV2 was shared by all 4 species (31.7% of *B. swezeyi* reads, 11.5% of *E. yasumatsui* reads, 36.8% of *S. nigricornis* reads, and 16.1% of *T. machilicola* reads), and 100% identical to *Arsenophonus* symbionts detected in various insect lineages, including aphids, whiteflies, louse flies, and the psyllid species *Cardiaspina tenuitela* (Aphalaridae: Spondyliaspidinae) (KY428657) and *Glycaspis brimblecombei* (Aphalaridae) (EU043378). In addition to SV2, one more distinct SV each for *Arsenophonus* was observed in these psyllids (SV29: 6.7% of *B. swezeyi* reads, SV27: 6.1% of *E. yasumatsui* reads, SV19: 13.4% of *S. nigricornis* reads, and SV44: 2.9% of *T. machilicola* reads). These SVs were 98.8% (SV27)–99.8% (SV2) identical to *Arsenophonus nasoniae* (CP038613), the type species of *Arsenophonus* found in the parasitoid wasp *Nasonia vitripennis* (Hymenoptera: Pteromalidae), and 97.9% (SV27)–98.8% (SV2) identical to “*Ca.* Arsenophonus triatominarum” (DQ508185) found in the assassin bug *Triatoma rubrofasciata* (Hemiptera: Reduviidae). These sequences formed a robustly supported clade (bootstrap: 100%) in the ML tree (Fig. 4). Although *Arsenophonus* shows a wide range of associations from parasitic to obligately mutualistic to host insects (Nováková *et al.*, 2009), its ecological role in psyllids currently remains unknown.

### *S. symbiotica* and its relatives

Four SVs found in *Epitrioza mizuhonica* and one in *E. yasumatsui* corresponded to the sequence of *S. symbiotica*, known as a prevalent secondary symbiont of aphids (Perreau *et al.*, 2021) (Fig. 1). SV22, SV23, SV26, and SV37, which accounted for 13.3, 11.6, 10.1, and 2.2%, respectively, of *E. mizuhonica* reads, and SV14, accounting for 18.5% of *E. yasumatsui* reads, were 98.6–99.5% identical to a single sequence of *S. symbiotica* (*e.g.* AB522706) (Fig. 4 and Supplementary Table S2). This reference sequence was derived from various aphid lineages, including *Acyrthosiphon pisum*, *Aphis fabae*, *Aphis gossypii*, *Cinara pinikoraiensis*, *Cinara ponderosae*, and *Trama caudata* (all Aphididae). The SVs described above and *S. symbiotica* sequences from aphids formed a robustly supported clade (bootstrap: 97%) together with SVs previously detected in another psyllid species *Cacopsylla coccinea* (Psyllidae: Psyllinae) (Nakabachi *et al.*, 2022) (Fig. 4). This result supports the recently proposed hypothesis that *S. symbiotica* is prevalent in psyllids (Nakabachi *et al.*, 2022), although it was formerly recognized only as a secondary symbiont of aphids. Since *S. symbiotica* was shown to enter plants and cause new infections in aphids, host plants are likely media for the intra- and inter-specific horizontal transmission of this bacterium (Perreau *et al.*, 2021).

SV22, SV23, and SV26 were 98.6% (SV23 vs SV26)–99.3% (SV22 vs SV23) identical to one another. Similarities in nucleotide sequences and read frequencies (Fig. 1 and Supplementary Table S2) implied that SVs corresponded to multiple copies of the 16S rRNA gene in a single *S. symbiotica* genome. This is consistent with a previous finding showing that the genomes of several *S. symbiotica* strains encoded more than a single copy of the 16S rRNA gene (Perreau *et al.*, 2021), which is in contrast to primary symbionts with an extremely streamlined genome encoding only a single copy (Nakabachi *et al.*, 2006, 2013b, 2020b; Moran *et al.*, 2008). A similar case was previously observed in *C. coccinea* described above (Nakabachi *et al.*, 2022). Although the ecological role of *S. symbiotica* widely varies depending on aphid lineages (Perreau *et al.*, 2021), its role in psyllids is currently unknown and, thus, warrants further study.

Additionally, the present study detected other lineages that are closely related to *S. symbiotica*. Five more SVs (SV20 from *E. mizuhonica*, SV21, SV28, SV42, and SV43 from *E. yasumatsui*) joined the above-described robustly supported clade with *S. symbiotica* (Fig. 4). However, we refrained from referring to the corresponding bacteria as *S. symbiotica* because their sequence identity with *S. symbiotica* was below the generally used arbitrary species threshold of 97% (94.6–96.5% for SV20, SV28, and SV43: *Serratia* endosymbionts) or genus threshold of 94.5–95% (92.1–92.3% for SV21 and SV42: Enterobacterales endosymbionts) (Yarza *et al.*, 2014; Barco *et al.*, 2020).

### Other *Enterobacterales* symbionts with uncertain identities

SV34, which accounted for 2.3% of *S. nigricornis* reads (Supplementary Table S2), was closely related to *Sodalis* endosymbionts (Fig. 4). It was 93.7% identical to the sequence of the type species *Sodalis glossinidius* (AP008232), a secondary symbiont of the tsetse fly *Glossina morsitans* (Diptera: Glossinidae). The sequence was 92.7–93.9% identical to those of *Sodalis* endosymbionts from various insects, including the other psyllid species *Cacopsylla burckhardti* (TAAB01000016), *C. kiushuensis* (Psyllidae: Psyllinae) (TAAB01000030), and *Cardiaspina maniformis* (Aphalaridae: Spondyliaspidinae) (KY428659). Although SV34 formed a moderately supported clade (bootstrap: 78%) with these sequences (Fig. 4), we refrained from assigning the corresponding bacterium to *Sodalis* and only referred to it as an *Enterobacterales* endosymbiont because its sequence identity was below the genus threshold (Yarza *et al.*, 2014; Barco *et al.*, 2020), and family names are fluid in *Enterobacterales* (Parks *et al.*, 2018). SV1 (62.7% of *T. camphorae* reads), SV33 (1.8% of *T. camphorae* reads), SV15 (63.2% of *T. machilicola* reads), SV3 (77.2% of *Pauropsylla triozoptera* reads), SV36 (2.0% of *P. triozoptera* reads), and SV4 (48.5% of *T. cinnamomi* reads) formed a cluster with a secondary endosymbiont of *Trioza magnoliae* (Triozidae) (AF077607) in the ML tree (Fig. 4). However, this branching pattern was only poorly supported (bootstrap: <50%), and their sequence identity with those of bacteria with a genus name was low (<94.5%). Therefore, the bacteria for these SVs were also named *Enterobacterales* endosymbionts (Fig. 4). SV11 (27.8% of *L. sulfurea* reads) deeply branched in the *Enterobacterales* tree (Fig. 4), and was closely related to “*Enterobacteriaceae*” bacteria, including symbionts of the other psyllid species *Epiacizzia kuwayamai* (Psyllidae: Macrocorsinae) (TAAB01000023) (86.5% identical), *C. biwa* (Psyllidae: Psyllinae) (TAAB01000001) (85.6% identical), and *C. kiushuensis* (Psyllidae: Psyllinae) (TAAB01000027) (85.6% identical). Since these sequence identities were above the arbitrary order threshold of 82.0%, but below the family threshold of 86.5% (Yarza *et al.*, 2014), the bacterium for SV11 was also referred to as an *Enterobacterales* endosymbiont (Fig. 4).

### *T. camphorae* has *Diplorickettsia*

**Fig. 5.**
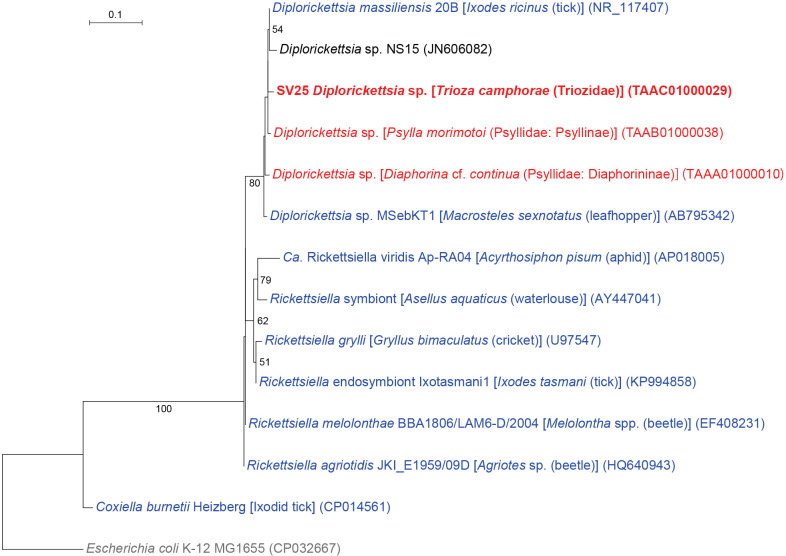
Phylogenetic position of *Diplorickettsia* lineages inferred by the maximum likelihood method. A total of 427 unambiguously aligned nucleotide sites of 16S rRNA genes were subjected to the ana­lysis. On each branch, bootstrap support values of >50% are shown. The scale bar indicates substitutions per site. Regarding symbiotic bacteria, host organisms are shown in brackets. Symbionts of animals other than psyllids are shown in blue. Symbionts of psyllids are shown in red. The sequence from the present study is shown in bold. DDBJ/EMBL/GenBank accession numbers are provided in parentheses. *Escherichia coli* was used as an outgroup.

Non-*Enterobacterales* gammaproteobacteria detected in the present study were *Carsonella* (*Oceanospirillales*: *Halomonadaceae*), the newly identified *Halomonadaceae* symbionts described above, and *Diplorickettsia* (*Diplorickettsiales*: *Diplorickettsiaceae*) (Fig. 1 and 5). SV25, which was derived from 7.0% of *T. camphorae* reads (Supplementary Table S2), was 98.8% identical to the sequence of *Diplorickettsia massiliensis* 20B (NR_117407), the type species detected in the European sheep tick *Ixodes ricinus* (Arachnida: Acari: Ixodidae), 98.6% identical to the sequence of *Diplorickettsia* sp. (TAAA01000038) recently found in the psyllid species *Psylla morimotoi* (Psyllidae: Psyllinae) (Nakabachi *et al.*, 2022), 97.9% identical to the sequence of *Diplorickettsia* sp. (TAAA01000010) found in the psyllid species *Diaphorina* cf. *continua* (Psyllidae: Diaphorininae) (Nakabachi *et al.*, 2020a), 97.9% identical to that of *Diplorickettsia* sp. MSebKT1 (AB795342) detected in the leafhopper *Macrosteles sexnotatus* (Hemiptera: Auchenorrhyncha: Cicadellidae), and 97.7% identical to *Diplorickettsia* sp. NS15 (JN606082) found in human nasal samples. In the ML tree, SV25 formed a well-supported clade (bootstrap: 80%) with these *Diplorickettsia* spp. (Fig. 5). *D. massiliensis* was observed in *I. ricinus* and patients with suspected tick-borne diseases, suggesting that this bacterium is a human pathogen (Subramanian *et al.*, 2012). *Diplorickettsia* lineages were then unexpectedly detected in the plant sap-sucking insects *M. sexnotatus* in Japan (Ishii *et al.*, 2013), *D.* cf. *continua* in Corsica (Nakabachi *et al.*, 2020a), and *P. morimotoi* in Japan (Nakabachi *et al.*, 2022). The present study adds another example of *Diplorickettsia* in Psylloidea. Collectively, the present results and previous findings suggest that *Diplorickettsia* is prevalent in various sap-sucking insects. Although their host plants are not shared among *M. sexnotatus* (Poaceae and Fabaceae), *D.* cf. *continua* (Thymelaeaceae), *P. morimotoi* (Rosaceae), and *T. camphorae* (Lauraceae), further studies are warranted to establish whether plants are also infected with *Diplorickettsia*. Since limited information is currently available on the functions of *Diplorickettsia* in host arthropods, the physiological and ecological effects of *Diplorickettsia* on psyllids need to be examined in more detail.

### First detection of *Liberibacter* in *E. yasumatsui*

Analyses detected “*Ca.* Liberibacter europaeus” (*C*Leu, *Alphaproteobacteria*: *Rhizobiales*) for the first time in *E. yasumatsui*, which feeds on *Elaeagnus umbellata* (Elaeagnaceae), known as the Japanese silverberry or autumn olive (Fig. 1 and Supplementary Table S2). SV17, which accounted for 16.9% of *E. yasumatsui* reads, was 100% identical to the sequence of *C*Leu previously detected in *C. pyri* (Psyllidae: Psyllinae) (FN678792) and *D.* cf. *continua* (Psyllidae: Diaphorininae) (TAAA01000007) (Nakabachi *et al.*, 2020a), and 99.8% identical to the sequence of *C*Leu recently detected in *Anomoneura mori* (Nakabachi *et al.*, 2022).

*C*Leu is a close relative of *C*Laf, “*Ca.* L. asiaticus”, and “*Ca.* L. americanus”, which are pathogens of the devastating greening disease in citrus (Rutaceae) (Grafton-Cardwell *et al.*, 2013), and *C*Lso, the pathogen causing serious diseases in solanaceous and apiaceous crops as described above (Mora *et al.*, 2021). *C*Leu was detected in various psyllids from various locations: *Cacopsylla* spp. (Psyllidae: Psyllinae) in Italy and Hungary, *Arytainilla spartiophila* (Psyllidae: Psyllinae) in New Zealand and the U.K. (Tannières *et al.*, 2020), *D.* cf. *continua* (Psyllidae: Diaphorininae) in Corsica island (Nakabachi *et al.*, 2020a), and *A. mori* in Japan (Nakabachi *et al.*, 2022). *C*Leu was also detected from rosaceous plants and the Scotch broom *Cytisus scoparius* (Fabaceae), which are host plants of *Cacopsylla* spp. and *Ar. spartiophila*, respectively (Tannières *et al.*, 2020). The presence of *C*Leu is associated with pathological symptoms in the Scotch broom (Tannières *et al.*, 2020). The present study adds another example of *C*Leu from the psyllid species *E. yasumatsui* in Japan. Further studies are required to clarify whether the host plant *E. umbellata*, which is distantly related to previously known infected plants, is also infected with *C*Leu and if infection causes disease symptoms. Since *E. umbellata* is an aggressive invasive plant in the United States and Europe, if *C*Leu causes a disease in *E. umbellata*, *C*Leu transmitted by *E. yasumatsui* may potentially be exploited as a biological herbicide.

### First detection of *Wolbachia* supergroup O in Psylloidea

**Fig. 6.**
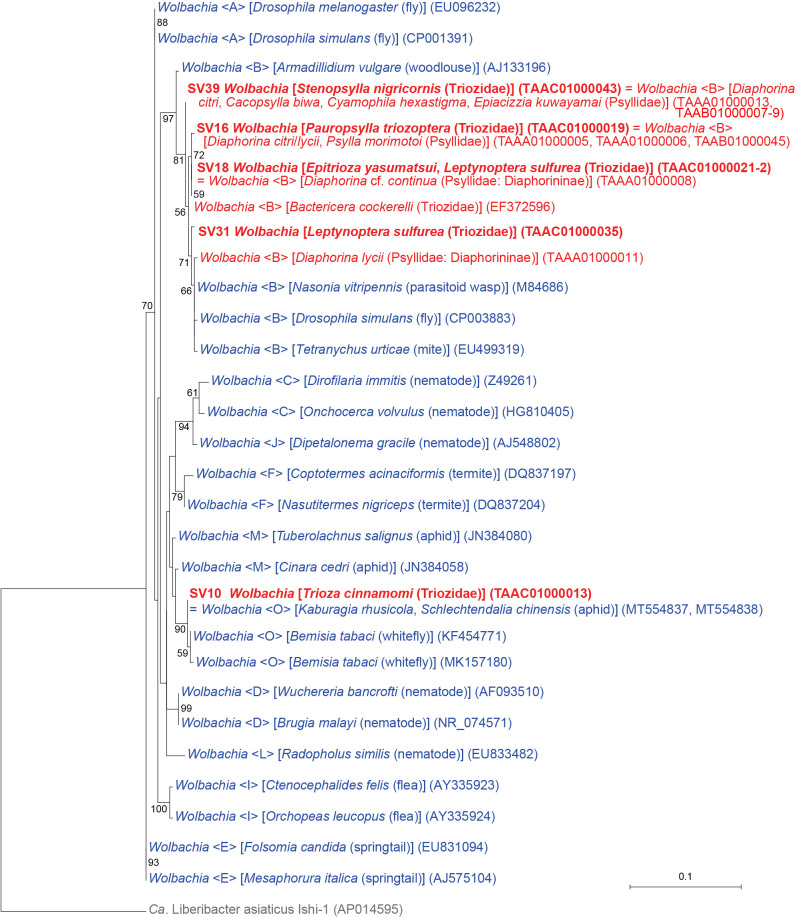
Maximum likelihood phylogram of *Wolbachia*. A total of 402 unambiguously aligned nucleotide sites of 16S rRNA genes were subjected to the ana­lysis. On each branch, bootstrap support values of >50% are shown. Host organisms are shown in brackets. Symbionts of animals other than psyllids are shown in blue. Symbionts of psyllids are shown in red. The sequence from the present study is shown in bold. DDBJ/EMBL/GenBank accession numbers for sequences are provided in parentheses. Supergroups of *Wolbachia* are shown in angle brackets. The scale bar represents nucleotide substitutions per position. *Liberibacter* was used as an outgroup.

Analyses identified five SVs corresponding to distinct lineages of *Wolbachia* (*Alphaproteobacteria*: *Rickettsiales*) (Fig. 1 and 6, Supplementary Table S2). *Wolbachia* are rickettsial bacteria that are distributed in a wide variety of arthropods and nematodes (Werren *et al.*, 2008), the strains of which are currently classified into supergroups A–Q (Lindsey *et al.*, 2016). Supergroups A and B are the most common supergroups infecting arthropods, and all *Wolbachia* strains previously detected in psyllids belonged to supergroup B (Spaulding and von Dohlen, 2001; Sloan and Moran, 2012; Arp *et al.*, 2014; Jain *et al.*, 2017; Morrow *et al.*, 2017; Chu *et al.*, 2019; Nakabachi *et al.*, 2020a, 2022). In contrast, SV10, which accounted for 25.9% of *T. cinnamomi* reads (Supplementary Table S2), was 100% identical to the sequence of *Wolbachia* belonging to supergroup O, which was detected in two aphid species, *Kaburagia rhusicola* (MT554837) and *Schlechtendalia chinensis* (MT554838) (Ren *et al.*, 2020). The ML ana­lysis placed the sequence within a robustly supported clade (bootstrap: 90%) of *Wolbachia* supergroup O (Fig. 6). To the best of our knowledge, this is the first detection of *Wolbachia* supergroup O in Psylloidea. All other *Wolbachia* strains found in the present study belonged to supergroup B (Fig. 6).

The majority of *Wolbachia* strains manipulate the reproduction of arthropod hosts to boost dissemination (Werren *et al.*, 2008). Due to this ability, *Wolbachia* are recognized as promising agents to control insect pests by affecting their traits or microbiomes (Brinker *et al.*, 2019). Based on the high infection rates of *Wolbachia* in pest psyllids worldwide (Spaulding and von Dohlen, 2001; Sloan and Moran, 2012; Arp *et al.*, 2014; Chu *et al.*, 2016, 2019; Morrow *et al.*, 2017; Nakabachi *et al.*, 2020a, 2022) and the suggested interactions between *Wolbachia* and other symbionts (Chu *et al.*, 2016, 2019; Jain *et al.*, 2017; Kruse *et al.*, 2017; Killiny, 2022), the application of *Wolbachia* to control pest psyllids and/or plant pathogens is anticipated (Chu *et al.*, 2016, 2019; Kruse *et al.*, 2017). The present results suggest the rampant horizontal transmission of various *Wolbachia* strains among various insects, including pest psyllids (Fig. 6); therefore, the artificial infection of *Wolbachia* appears to be feasible in psyllids and will facilitate the exploitation of this bacterial group as a tool to control pest psyllids and/or the plant pathogens they carry.

### *L. sulfurea* has an *Enterococcus* symbiont

**Fig. 7.**
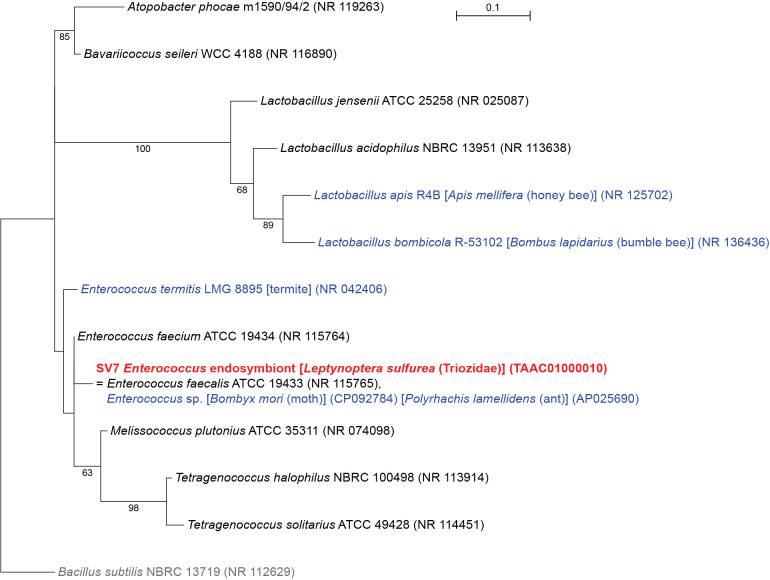
Maximum likelihood phylogram of bacteria belonging to the order *Lactobacillales*. A total of 428 unambiguously aligned nucleotide sites of 16S rRNA genes were subjected to the ana­lysis. On each branch, bootstrap support values of >50% are shown. Host organisms are shown in brackets. Bacteria found in insects other than psyllids are shown in blue. The sequence from the present study is shown in bold red. DDBJ/EMBL/GenBank accession numbers for sequences are provided in parentheses. The scale bar represents nucleotide substitutions per position. *Bacillus subtilis* was used as an outgroup.

SV7, which accounted for up to 40.0% of *L. sulfurea* reads (Supplementary Table S2), was 100% identical to the sequence of *Enterococcus faecalis* (*Firmicutes: Bacilli: Lactobacillales: Enterococcaceae*) found in mammalian animals (*e.g.* NR 115765), and *Enterococcus* spp. identified in marine sponges (*e.g.* MT484183) and insect lineages, including the silk moth *Bombyx mori* (CP092784), the ant *Polyrhachis lamellidens* (AP025690), and the flies *Bactrocera dorsalis* (MK764705) and *Hermetia illucens* (OK012249) (Akami *et al.*, 2019) (Fig. 7). Although *E. faecalis* is a common bacterium found in the gut of mammals, including humans, we consider this SV to be derived not from contaminants, but from a stable associate of *L. sulfurea* because 1) SV7 accounted for a large percentage of *L. sulfurea* reads, and 2) no other SVs in *L. sulfurea* were similar to the bacterial sequences found in the animal intestinal flora. Gram-positive *E. faecalis* (*Firmicutes*: *Bacilli*: *Lactobacillales*: *Enterococcaceae*), which is distantly related to all other Gram-negative proteobacterial symbionts identified in the present study, has only been recognized as a transient gut resident in insects (Akami *et al.*, 2019). Therefore, the localization and functional role of this bacterium in *L. sulfurea* need to be examined in more detail.

## Conclusions

The present study identified various bacterial symbionts in nine psyllid species of the family Triozidae. The majority of secondary symbionts were gammaproteobacteria, particularly those of the order *Enterobacterales*, including *Arsenophonus* and *S. symbiotica*. Regarding non-*Enterobacterales* gammaproteobacteria, *Diplorickettsia* (*Diplorickettsiales*: *Diplorickettsiaceae*), a potential human‍ ‍pathogen, and *Carnimonas* (*Oceanospirillales*: *Halomonadaceae*), a lineage independent of *Carsonella* within the family Halomonadaceae, were identified. As for alphaproteobacteria, the potential plant pathogen *C*Leu (*Rhizobiales*: *Rhizobiaceae*) was detected for the first time in *E. yasumatsui*. Since *E. yasumatsui* feeds on the Japanese silverberry *E. umbellata* (Elaeagnaceae), which is an aggressive invasive plant in the United States and Europe, the combination of *E. yasumatsui* and *C*Leu may potentially be exploited as a biological herbicide for *E. umbellata*. *Wolbachia* (*Rickettsiales*: *Anaplasmataceae*) strains of supergroup B were identified in three Triozidae species, whereas a lineage belonging to supergroup O was detected in *T. cinnamomi*, which is the first report of this supergroup in Psylloidea. Moreover, *E. faecalis* (*Firmicutes*: *Bacilli*: *Lactobacillales*: *Enterococcaceae*), which has only been recognized as a transient resident in insects, was suggested to constitute a large part of the microbiome in *L. sulfurea*, implying that this bacterium acquired the status of a stable symbiont. These results provide more detailed insights into the interactions among insects, bacteria, and plants, which may be exploited to facilitate the control of pest psyllids in the future.

## Citation

Nakabachi, A., Inoue, H., and Hirose, Y. (2022) High-resolution Microbiome Analyses of Nine Psyllid Species of the Family Triozidae Identified Previously Unrecognized but Major Bacterial Populations, including *Liberibacter* and *Wolbachia* of Supergroup O. *Microbes Environ ***37**: ME22078.

https://doi.org/10.1264/jsme2.ME22078

## Supplementary Material

Supplementary Material 1

Supplementary Material 2

## Figures and Tables

**Table 1.** Psyllid species used in the present study.

Species	Sampling site	Collection date	Host plant
*Baeoalitriozus swezeyi* (Crawford)	Kijoka, Ogimi, Okinawa Is., Ryukyus, Japan (26.705 N 128.145 E)	April 20, 2009	*Diospyros ferra* (Ebenaceae)
*Epitrioza mizuhonica* Kuwayama	Nishikawachi, Reihoku, Kumamoto Pref., Amakusa-shimoshima Is., Kyushu, Japan (32.538 N 130.106 E)	May 15, 2010	*Elaeagnus umbellata* (Elaeagnaceae)
*Epitrioza yasumatsui* Miyatake	Mitsu, Akitsu-chô, Higashihiroshima city, Hiroshima Pref., Honshu, Japan (34.330 N 132.823 E)	June 20, 2016	*Elaeagnus umbellata* (Elaeagnaceae)
*Leptynoptera sulfurea* Crawford	Kabira, Ishigaki Is., Ryukyus, Japan (24.461 N 124.143 E)	May 2, 2000	*Calophyllum inophyllum* (Calophyllaceae)
*Pauropsylla triozoptera* Crawford	Mt. Nekumatidi-dake, Ogimi, Okinawa Is., Ryukyus, Japan (26.682 N 128.138 E)	April 20, 2009	*Ficus ampelas* (Moraceae)
*Stenopsylla nigricornis* (Kuwayama)	Nobeoka city, Miyazaki Pref., Kyushu, Japan (32.563 N 131.613 E)	March 24, 2000	*Symplocos kuroki* (Symplocaceae)
*Trioza camphorae* Sasaki	Ideno, Minamihata, Imari city, Saga Pref., Kyushu, Japan (33.313 N 129.929 E)	April 5, 2012	*Cinnamomum camphora* (Lauraceae)
*Trioza cinnamomi* (Boselli)	Hayasaki, Kuchinotsu-chô, Minamishimabara city, Nagasaki Pref., Kyushu, Japan (32.600 N 130.186 E)	April 23, 2015	*Cinnamomum tenuifolium* (Lauraceae)
*Trioza machilicola* Miyatake	Koba, Obama-chô, Unzen city, Nagasaki Pref., Kyushu, Japan (32.698 N 130.227 E)	April 30, 2015	*Machilus thunbergii* (Lauraceae)
